# Structural modification and biological activities of carboxymethyl *Pachymaran*

**DOI:** 10.1002/fsn3.2404

**Published:** 2021-06-30

**Authors:** You‐Yu Yan, Shuai Yuan, Hao‐Hai Ma, Xi‐Feng Zhang

**Affiliations:** ^1^ School of Life Science and Technology Wuhan Polytechnic University Wuhan China; ^2^ College of Veterinary Medicine Qingdao Agricultural University Qingdao People’s Republic of China

**Keywords:** antitumor, polysaccharide iron, polysaccharide selenium, polysaccharide zinc, polysaccharides, *Poria cocos*

## Abstract

Polysaccharides are good chelating agents for metal ions, which are often used to synthesize polysaccharide metal ion complexes. With carboxymethyl *pachymaran* (CMP) as the substrate, carboxymethyl *pachymaran* iron (CMPF), carboxymethyl *pachymaran* selenium (CMPS), and carboxymethyl *pachymaran* zinc (CMPZ) were synthesized by response surface methodology, and their biological characteristics were studied. The results showed that the CMP was a β‐polysaccharide, and the degree of carboxymethylation was 0.6352. The polysaccharide metal ion complexes were characterized by physicochemical methods, scanning electron microscopy, Fourier transform infrared spectroscopy, circular dichroism spectroscopy, and nuclear magnetic resonance spectroscopy. All the polysaccharides and complexes possessed antioxidant activity in vitro with scavenging activities to ABTS, superoxide anions, and ferrous ions. CMPF, CMPS, and CMPZ caused significant inhibition of A2780 cell proliferation, promoted the production of reactive oxygen species, and induced apoptosis in a human ovarian cancer cell line (A2780 cells). These results suggest that the CMP complex may be an effective candidate drug for cancer treatment in the field of functional food and pharmacology.

## Graphical abstract

Carboxymethyl *pachymaran* as the substrate, the carboxymethyl *pachymaran* iron, carboxymethyl *pachymaran* selenium, and carboxymethyl *pachymaran* zinc were synthesized by response surface methodology, and their biological characteristics were studied.

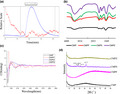

## 1 INTRODUCTION

*Poria cocos*, a dual‐purpose fungus from China, has both medicinal and edible properties. It confers good disease resistance, is safe, and has no toxicity. It has attracted the attention and research of scientific and technological workers in many fields, such as food science, medicine, pharmacy, and biology (Liu, Yin, et al., 2019; Liu, Liu, et al., 2019; Xu et al., 2019). *Poria cocos* has a long history of good curative effects in China. It can be used as a Chinese herbal medicine with many other herbal medicines to form a prescription to enhance its efficacy. Therefore, *P. cocos* is a very important Chinese herbal medicine, and can also be used for health care and consumption (Sun et al., 2019; Wang et al., 2020; Zhang, Zhu, et al., 2019; Zhang, Khan, et al., 2019). *Poria cocos* contains a variety of active components, the main components of which are polysaccharides and terpenes (Li et al., 2019; Sun et al., 2019). Plant polysaccharides and their complexes participate in the regulation of various vital activities in cells. Natural polymer polysaccharides have many pharmacological activities and no toxic side effects on normal cells, which has increased research into using polysaccharides to treat related diseases. Modern research shows that polysaccharides isolated from plants have many biological activities, such as antitumor, immune regulatory, antioxidant, anti‐inflammatory, antiviral, and hypoglycaemic activity (Cho et al., 2015; Rjeibi et al., 2019; Zhan et al., 2019).

Polysaccharide is a kind of natural polymer containing a ketone group or an aldehyde group, and is composed of more than 10 monosaccharides linked by glycosidic bonds. They are widely found in animals, plants, and microorganisms, and are another important biological macromolecule besides nucleic acids and proteins. Polysaccharides are polymers made of different monosaccharides and various structures, which leads to different properties and activities of polysaccharides in different plants. As early as 1971, Hoffmann discovered the β‐(1→3)‐D‐glucopyranosyl component of the β‐(1→6) branched‐chain in *P. cocos* polysaccharide (Hoffmann et al., 1971), and Kanayama found β‐(1→3)‐(1→6)‐D‐glucan in polysaccharides (Kanayama et al., 1983). Later, Zhang et al. (2003) found that the *P. cocos* polysaccharide is composed of β‐(1→3)‐D‐glucan and α‐(1→3)‐D‐glucan; it also contains a small number of branches a D‐glucuronic acid β‐(1→3)‐d‐glucan. Furthermore, Ruidian performed detailed tests on *P. cocos* polysaccharide and found that it is composed of ribose, arabinose, xylose, mannose, glucose, and galactose (Ke et al., 2010).

Metal ions can react with the hydroxyl and carboxyl groups of polysaccharides to form complexes or esters (Liao et al., 2015; Liu, Yin, et al., 2019; Liu, Liu, et al., 2019; Moe et al., 2019). Polysaccharide zinc, polysaccharide selenium, and polysaccharide iron enhance the inhibition mediated by polysaccharides on hepatoma cells, breast cancer cells, and colon cancer cells (Deng et al., 2009; Ding et al., 2019; Liao et al., 2015; Shang et al., 2009). At the same time, compared with inorganic metal patches with considerable side effects, organic metal patches have fewer side effects.

In this study, response surface methodology was used to synthesize carboxymethyl *pachymaran* iron (CMPF), carboxymethyl *pachymaran* selenium (CMPS), and carboxymethyl *pachymaran* zinc (CMPZ), and the antioxidant effects of CMPF, CMPZ, and CMPS were investigated. The human ovarian cancer cell line A2780 was used as a model cell line to investigate the anticancer activity of these compounds.

## 2 MATERIALS AND METHODS

### 2.1 Drugs and reagents

All the chemicals and solvents used in this study were of analytical grade.

### 2.2 Preparation of CMP

The preparation method was modified with reference to those of Zhang and Wang (2006) by one‐step carboxylation of *P. cocos* powder and chloroacetic acid in an alkaline environment. CMP was obtained after protein being removed, alcohol precipitation and dialysis. The content of polysaccharides was determined by the sulfuric acid phenol method, and the degree of substitution of carboxymethylation in CMP was determined by the titration method, which was modified according to the published methods. The specific measurement method was as follows: 0.01 g of CMP was accurately weighed and was put into a 100 ml conical flask, and then, 3 ml of 70% ethanol was added into and mixed until uniformly for being allowed to stand for 5 min. Subsequently, 50 ml of 0.5 mol/L NaOH and 10 ml of pure water were added and completely dissolved, then the pH of which was adjusted to 7.0 with 0.1 mol/L hydrochloric acid, where phenolphthalein was used as the indicator. The formula for the number of millimoles (a) of hydrochloric acid required for each gram of CMP in pH regulation is as follows:

$$A=\frac{V_{0}M_{0}‐\frac{\left(V_{2}‐V_{1}\right)}{M}}{m}$$

In the above equation: *V*_0_: volume of sodium hydroxide added (ml), *V*_2_: volume of hydrochloric acid required for sample determination (ml), *V*_1_: volume of hydrochloric acid required for blank determination (ml), *M*_0_: concentration of sodium hydroxide (0.5 mol/L), *M*: concentration of hydrochloric acid (0.1 mol/L), and *m*: quantity of sample (g).

The degree of carboxymethylation substitution (Ds) of CMP was calculated according to the following formula:

$$\text{Ds}=\frac{0.162A}{1‐0.058A}$$

### 2.3 Determination of molecular weight

The CMP molecule was measured with HPSEC‐MALLS‐RID on the basis of the dn/dc method according to previous reported methods (Yao et al., 2020). A HPSEC columns (TSKgel SuperMultipore PW‐M, Tosoh), MALLS detector (DAWN HELEOSII, Wyatt Technology), and refractive index detector (2414, Waters) integrated to the HPSEC‐MALLS‐RID.

### 2.4 Synthesis of CMPF

**CMPF is synthesized** according to the method of preparing polysaccharide iron by Wang et al. (2014), the specific steps of which were as follows: a proper amount of CMP was weighed into a round bottom flask, then a certain amount of trisodium citrate and pure water were added to completely dissolve the CMP. Under alkaline conditions of sodium hydroxide, prepared ferric chloride was added drop by drop under agitation, stopped immediately until there was precipitation, then the pH was adjusted, and the reaction solution was heated in a water bath for a certain period of time. Subsequently, the reaction solution was centrifuged at 7,104 *g*/min for 5 min, then the supernatant was moved into a conical flask, and a red‐brown precipitate was obtained by adding three times the volume of alcohol at 4℃ overnight to yield CMPF. Finally, the precipitate was washed three times with anhydrous ethanol. After dissolution, the salt ions were removed by dialysis, and then, the CMPF was obtained by freeze‐drying (Data S1).

### 2.5 Synthesis of CMPS

According to a published method (Gao et al., 2012), an appropriate amount of CMP was dissolved in dilute nitric acid, then appropriate sodium selenite and the catalyst barium chloride were added into and was heated for a certain time, cooled to room temperature after the reaction, adjusted to pH 7 with sodium carbonate, and then, sodium sulfate was added to make barium ions precipitate. Centrifugation was done at 7,104 *g*/min for 5 min, soon afterward the supernatant was collected finally the white precipitate, CMPS, was obtained by adding three times the volume of alcohol for precipitating in a refrigerator at 4℃ overnight; the precipitate was further washed three times with anhydrous ethanol. After reconstitution, the salt ions were removed by dialysis, and then, the CMPS was obtained by freeze‐drying (Data S2).

### 2.6 Synthesis of CMPZ

According to a published method (Dong et al., 2018), a proper amount of CMP was weighed into a round bottom flask, and water was added to make it dissolve with a certain amount of zinc acetate added slowly; then, the pH was adjusted to 6–7, and the reaction solution was heated in a water bath for a certain period of time. After the reaction was completed, the sample was centrifuged at 8,000 rpm/min for 5 min and the supernatant was put into a conical flask; subsequently, the white precipitate, CMPZ, was obtained by adding three times the volume of alcohol in a refrigerator at 4℃ overnight, which was washed three times with anhydrous ethanol. After dissolution, the salt ions were removed by dialysis; finally, the CMPZ was obtained by freeze‐drying (Data S3).

### 2.7 Characterization of CMPF, CMPS, and CMPZ

#### 2.7.1 FTIR analysis

Approximately 2 mg of CMPF, CMPS, and CMPZ powder were weighed and mixed with KBr powder, then ground and pressed for fourier transform infrared (FTIR) measurement through spectrometry (PerkinElmer, Spectrum 400).

#### 2.7.2 CD

The concentration of polysaccharide was 0.5 mg/ml. Determination conditions were as follows: temperature: 25℃, scanning wavelength range: 185–300 nm, resolution: 0.2 nm, response time: 0.25 s, scanning rate: 100 nm/min. Chirascan V100 CD (Applied Photophysics) spectropolarimeter was used for circular dichroism (CD) analysis.

#### 2.7.3 XRD

A certain amount of samples were scanned in the sample room of X‐ray diffractometer (D8ADVANCE, Bruker). The scanning range was 5° to 90°, and X‐ray irradiation was carried out at the rate of 5°/min. The XRD diffraction intensity curve was drawn with the obtained data.

#### 2.7.4 SEM

The sample table was placed in the ion sputtering apparatus and coated with a layer of conductive gold powder, to which a proper amount of sample was adhered with copper tape. Then, the samples were placed under a scanning electron microscope (Hitachi) for observation. Working conditions were as follows: The accelerating voltage was 15 kV, and magnifications were 500×, 1000×, and 2000×. The corresponding definition was adjusted until the ideal visual field was obtained, and the appropriate visual field was selected to take photographs. Each sample was imaged three times to eliminate sample interference and system error.

#### 2.7.5 TGA

A 0.5 mg of sample was weighed and used for thermogravimetric analysis under a simultaneous thermal analyzer (STA449F3, Netzsch). The corresponding parameters were as follows: Nitrogen was used as the carrier gas, the flow rate of which in the system was 20 mM/min, the rate of temperature rise was 10℃/min, and the temperature range was 30–650℃.

#### 2.7.6 NMR analysis

A Bruker AV‐400 nuclear magnetic resonance (NMR) spectrometer (Bruker Instrumental Inc.) was used to record the ^1^H NMR spectra of the CMP, CMPF, CMPS, and CMPZ at 25℃, which were dissolved in deuteration DMSO.

### 2.8 Antioxidant activities

The antioxidant activities of CMP, CMPF, CMPS, and CMPZ were assessed on basis of previously established methods (Du et al., 2013; Jeddou et al., 2016). For this, 1, 2, 4, 8, and 10 mg/ml of CMP, CMPF, CMPS, and CMPZ were dissolved in water. The scavenging ability was calculated by the following equation: $\text{inhibition rate}\;\left(\%\right)\;=\;\left(1‐\frac{A_{1}‐A_{2}}{A_{0}}\times 100\%\right)$, where $A_{0}$ is the absorbance of the control (water instead of sample),$A_{1}$ is the absorbance of the sample, and $A_{2}$ is the absorbance of the sample with anhydrous ethanol instead of pyrogallol.

### 2.9 Cytotoxicity assay of CMP, CMPF, CMPS, and CMPZ

A2780 cells were cultured in DMEM/F12 medium + FBS (10%) and antibiotics (100 U/ml penicillin together with 100 µg/ml streptomycin) in a 5% CO_2_ atmosphere at 37℃ in a humidified incubator. Different concentrations of CMP, CMPF, and CMPS (0, 100, 200, 300, 400, and 500 μg/ml) were used, respectively, to treat cells for 24 hr, and so was CMPZ (0, 20, 40, 60, 80, and 100 μg/ml) for 6 hr. An 3‐(4,5)‐dimethylthiahiazo (‐z‐y1)‐3,5‐di‐phenytetrazoliumromide (MTT) assay was used to evaluate the cytotoxicity of CMP, CMPF, CMPS, and CMPZ.

### 2.10 ROS assays and TUNEL assay

An S0033 detection kit (Beyotime) was used to measure intracellular reactive oxygen species (ROS) levels in accordance with the instructions. An MA0233 detection kit (Meilum one‐step terminal deoxyribonucleotide transferase‐mediated nick end labeling [TUNEL] apoptosis assay kit [FTIC], Melonepharma) was used to measure the TUNEL levels in accordance with the included instructions.

### 2.11 Statistical method

Values were statistically tested using Student's *t* test or one‐way analysis of variance, followed by the Tukey test for multiple comparisons. Comparisons were considered significant at *p* < .05 and *p* < .01 (asterisk).

## 3 RESULTS AND ANALYSIS

### 3.1 Molecular weight

**FIGURE 1:**
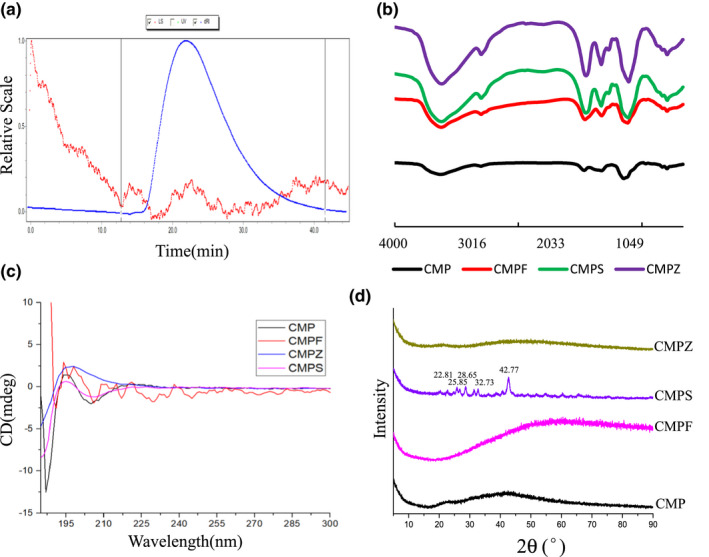
Spectroscopic characterization of CMP, CMPF, CMPS, and CMPZ. (a) Molecular weight of CMP determination with HPSEC‐MALLS‐RID system. FTIR spectra. (b) CD spectra. (c) XRD assay of CMP, CMPF, CMPS, and CMPZ

The molecular weight of CMP was determined by HPSEC‐MALLSRID. The differential detection signal (dRI) of CMP showed one peak at about 16–34 (Figure 1a). The weight average molecular weight (Mw) was 3.759 × 10^5^ g/mol and the number average molecular weight (Mn) was 3.689 × 10^5^ g/mol. The Mw/Mn of peak was 1.019, which indicated the molecular weight distribution of CMP was relatively concentrated.

### 3.2 Spectroscopic characterization of synthesized complexes

#### 3.2.1 FTIR assay

**FIGURE 2:**
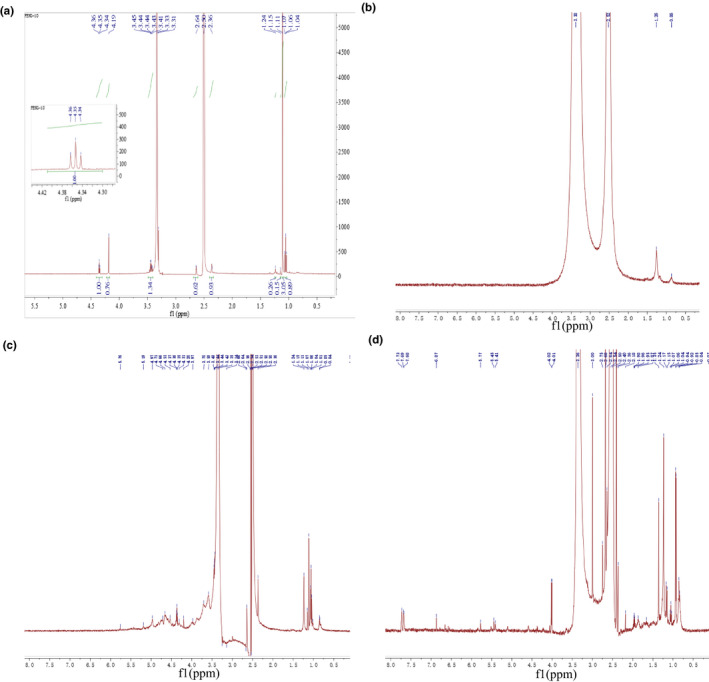
The ^1^H spectra of CMP, CMPF, CMPS, and CMPZ. (a) The ^1^H spectra of CMP. (b) The ^1^H spectra of CMPF. (c) The ^1^H spectra of CMPS. (d) The ^1^H spectra of CMPZ

As shown in Figure 2, the prepared CMP sample shows a very typical characteristic absorption peak of polysaccharide. The absorption peak between 3,600 and 3,200 cm^−1^ is wide, which is caused by the stretching vibrations of the non‐free O‐H bond of the sugar chain, while the absorption peak of 2,928.66 cm^−1^ is caused by the stretching vibrations of glycomethyl and the methylene C‐H bond. The peaks at 1636.95 and 1,422.09 cm^−1^ are the characteristic absorption peaks of carboxymethyl, which are generated by the asymmetric stretching vibrations of C=O and methylene stretching vibrations, respectively. In addition, the peak near 869.07 cm^−1^ is the characteristic absorption peak of β‐pyranose. There is no characteristic absorption peak at 810 and 860 cm^−1^ in the infrared spectrum, indicating that the CMP component does not contain mannose.

The infrared spectra of CMPF, CMPZ, and CMPS are shown in Figure 1a. It can be seen that the basic configuration of the polysaccharide had not changed greatly after the complexation of CMP with iron ions, indicating that the basic structure of the polysaccharide had not been destroyed. The peak ‐OH stretching vibrations of polysaccharide changed from 3,432.17 to 3,402.22 cm^−1^ of CMPF, which shifted to the direction of lower wavenumber, and the peak of the ‐OH stretching vibrations of the polysaccharide iron was narrower compared with that of the polysaccharide, indicating that the ‐OH of the polysaccharide participated in complexation. At the same time, the C=O stretching vibration peak of the polysaccharide at 1636.95 cm^−1^ turned into 1602.04 cm^−1^ of the polysaccharide iron, which also shifted to the direction of lower wavenumbers, indicating that C=O also participated in the complexation of iron. According to the comparison of the infrared spectrum, the preparation of polysaccharide iron was successful. A polysaccharide is a polymer with various structures, but the molecular structure of polysaccharide iron has not been determined. Chen and Wang (2008) systematically summarized the possible structure of polysaccharide iron by X‐ray powder diffraction. The iron atoms are in two DxCOOH ligands, and the other seven DxCOOH exist in a random spiral around the iron core. The carboxyl end is covalently linked to the iron core, while the other side is hydrogen‐bonded to the iron core. The iron core exists in the form of β‐FeOOH by light diffraction and the Mossbauer spectrum (Coe et al., 1995).

The ‐OH stretching vibration peak of the polysaccharide changed from 3,432.17 to 3,405.84 cm^−1^ in CMPZ, which shifted to the direction of lower wavenumbers, indicating that the ‐OH of the polysaccharide participated in the complexation. At the same time, the C=O stretching vibration peak of polysaccharide at 1636.95 cm^−1^ turned into 1616.14 cm^−1^ in the polysaccharide zinc, which also shifted in the direction of lower wavenumbers, indicating that the C=O also participated in zinc complexation. According to the comparison of the infrared spectrum, the preparation of polysaccharide zinc was successful. The absorption peak of the carboxyl group in CMP was 1636.95 cm^−1^, while the absorption peak of CMPS shifted to 1,600.71 cm^−1^ with a lower wavenumber, so the characteristic absorption peak of the carboxyl group changed, indicating that the carboxyl group at the C_2_ position had a chemical reaction to weaken its bond force. In addition, the stretching vibration peak of C‐O of CMP shifted from 1,143.96 to 1,076.14 cm^−1^ of CMPS, indicating that the hydroxyl group on C_6_ also participated in the reaction.

#### 3.2.2 CD analysis

As shown in Figure 1b, when the wavelength shifted from long waves to the short waves, the CD spectra of CMP and CMPF aqueous solutions changed from peak to valley, showing a positive Cotton effect. The CD spectra of CMPS and CMPZ aqueous solutions changed from valley to peak, showing a negative Cotton effect. The different wavelengths of valleys and peaks indicate that they have different conformations and are different substances.

#### 3.2.3 XRD analysis

The results of XRD analysis of the samples (Figure 1c) show that samples of CMP, CMPF, and CMPZ had no obvious dominant absorption peak in the range of 5–90°, indicating the three samples cannot form single crystal, and are irregular and amorphous. The peaks of XRD intensity curves of CMP, CMPF, and CMPZ appear at 2θ of 42°, 58°, and 46°, respectively, and the peak shape is relatively flat, which indicates that the crystallinity is low. CMPS showed new characteristic peaks at 22.81°, 25.85°, 28.65°, 32.73°, and 42.77° with strong signals, which indicated that CMPS was more prone to single crystal morphology, and new chemical structure substances were formed after chemical synthesis of Na_2_SeO_3_ and CMP.

#### 3.2.4 ^1^H NMR assay

As shown in Figure 2a, the ^1^H NMR spectrum of CMP shows that there is a triple peak of cephalic hydrogen at δ 4.34, δ 4.35, and δ 4.36 ppm in the chemical shift δ 4.3–5.9 ppm, which indicates that CMP exists in the form of β‐pyranose, which is also consistent with the research of Saito (Hiroshi et al., 1968). However, the complete signal pattern of CMP was missing in the spectrum of CMPF (Figure 2b), whether in its usual range or shifted, which is similar to the results of Wang (Wang et al., 2015). Figure 2c is the ^1^H NMR of CMPS, the signal distribution is mainly concentrated in the range of δ 3.0–5.5 ppm (5.19, 4.97, 4.71, 4.66, 4.53, 4.37, 4.36, 4.35, 4.31, 4.20, 3.97, 3.7, 3.59,3.45, 3.44, 3.43, 3.35, 3.14), which is a typical polysaccharide signal peak. The signal distribution of CMPZ is mainly concentrated in the range of δ 2.0–4.5 ppm and δ 2.0–4.5 ppm and δ 0.8–1.2 ppm (Figure 2d).

#### 3.2.5 SEM analysis

**FIGURE 3:**
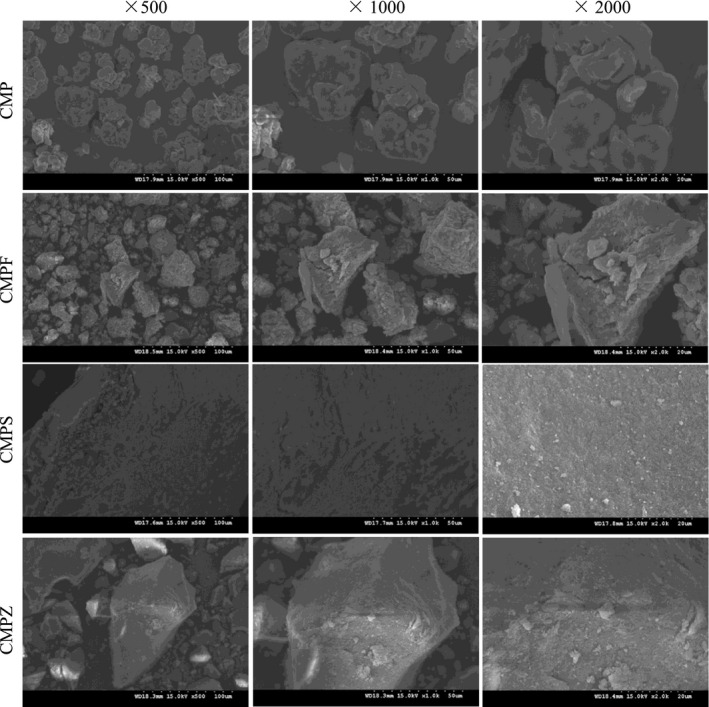
SEM images of CMP, CMPF, CMPS, and CMPZ

Scanning electron microscopy (SEM) was used as a qualitative tool to analyze the surface morphology of the polysaccharides. The microphotographs show the morphology of four fragmented samples (Figure 3). The SEM CMP and CMPF showed as cheese‐like lumps, and the surface of the fragments was slightly uneven. CMPZ was granular with a rough surface. CMPS had a dense lamellar structure, smooth surface, uniform texture, and a small amount of particles. The interaction between selenium and polysaccharide changed the relationship between that of molecules and van der Waals forces, which indicated that the enrichment of selenium had a considerable influence on the structure of CMP, resulting in an increase in the intermolecular force and the degree of cross‐linking of polysaccharides, which led to the change of the appearance of CMP aggregates.

#### 3.2.6 TGA analysis

**FIGURE 4:**
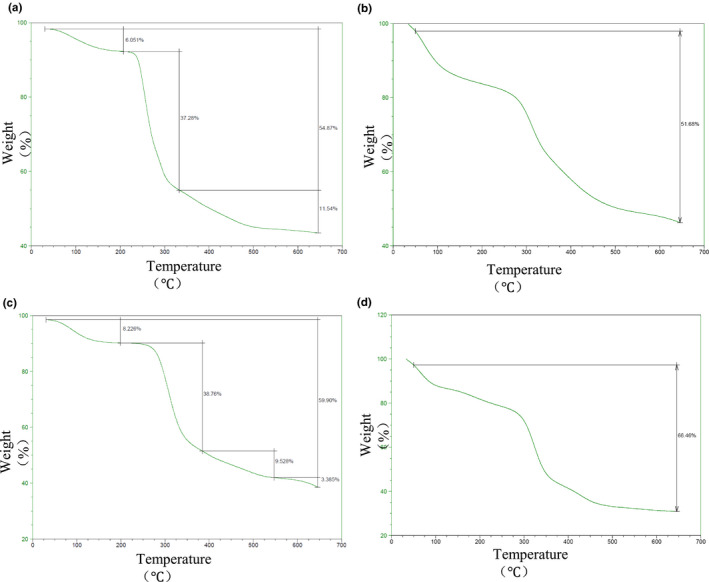
TGA curves of CMP, CMPF, CMPS, and CMPZ under nitrogen conditions. (a) TGA curves of CMP. (b) TGA curves of CMPF. (c) TGA curves of CMPS. (d) TGA curves of CMPZ

It can be seen from Figure 4 that all the four samples underwent the weight loss phenomenon. The weight loss of CMP and CMPS was relatively gentle at low temperatures, and sharp when the temperature was higher than 200℃. CMPF and CMPZ showed considerable weight loss at low temperatures, which may be caused by the loss of adsorbed water; the weight loss was relatively gentle at temperatures higher than 400℃. In general, CMPZ had more weight loss than the other three samples (Figure 4d).

### 3.3 Antioxidant activity of CMP, CMPF, CMPS, and CMPZ

**FIGURE 5:**
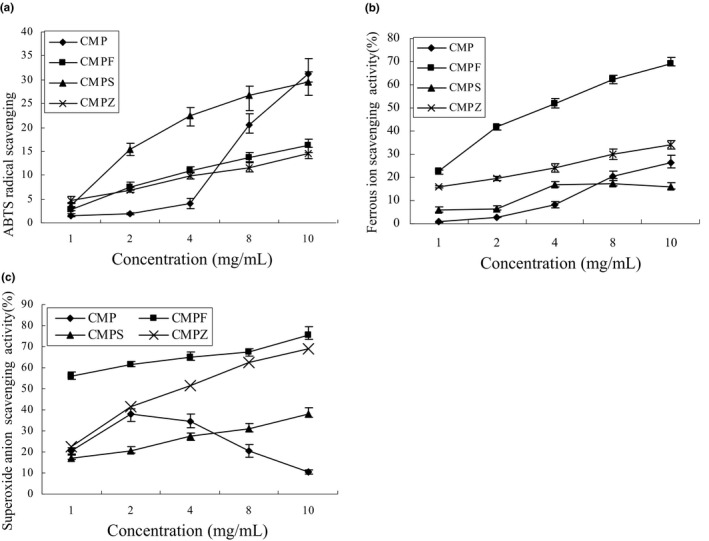
Antioxidant activity of CMP, CMPF, CMPS, and CMPZ (a) Scavenging effects of CMP, CMPF, CMPS, and CMPZ on ABTS radicals. Values are means ± SD (n = 3). (b) Scavenging effects of CMP, CMPF, CMPS, and CMPZ on ferrous ion radical scavenging rate. Value are means ± SD (n = 3). (c) Scavenging effects of CMP, CMPF, CMPS, and CMPZ on superoxide ion radicals. Values are means ± SD (n = 3)

As shown in Figure 5a, the ABTS radical scavenging activity of CMP, CMPF, CMPS, and CMPZ increased with an increase in concentration. At the concentration of 10 mg/ml, the scavenging ability of ABTS radicals reached the maximum values, 31.22%, 16.22%, 29.61%, and 14.59%, respectively. After modification, the scavenging ability to ABTS radicals of CMPF, CMPS, and CMPZ was lower than that of CMP.

As shown in Figure 5b, the ferrous ion scavenging activity of CMP, CMPF, and CMPZ increased with the increase in concentration, only the ferrous ion scavenging activity of CMPS first increased and then decreased with the increase in concentration. The maximum ferrous ion scavenging ability of CMPF and CMPZ was higher than that of CMP, and the ability of CMPS was lower than that of CMP. The maximum ferrous ion scavenging ability of CMP, CMPF, CMPS, and CMPZ was 26.44%, 69.2%, 16.09%, and 34.04%, respectively.

As shown in Figure 5c, the superoxide anion scavenging activity of CMP first increased and then decreased with an increase in the polysaccharide concentration, with a maximum scavenging rate of about 40%. Compared with that of CMP, the superoxide anion scavenging activity of CMPF and CMPZ was significantly higher, and the maximum superoxide anion scavenging activity of CMPF and CMPZ was 75.68% and 69.2%, respectively. The maximum superoxide anion scavenging activity of CMPS was 37.93% at the concentration of 10 mg/ml.

Oxidative stress is considered to be the imbalance between oxidants and antioxidants in the body. It is usually eliminated by the antioxidant defense system. Excessive free radicals are inhibited by the antioxidant defense system to prevent tissue damage and disease. Antioxidants from natural sources have strong antioxidant activities in scavenging free radicals and reducing cell damage caused by oxidation and have been added to health products, food additives, and drugs.

### 3.4 Effect of CMP, CMPF, CMPS, and CMPZ on cell viability

**FIGURE 6:**
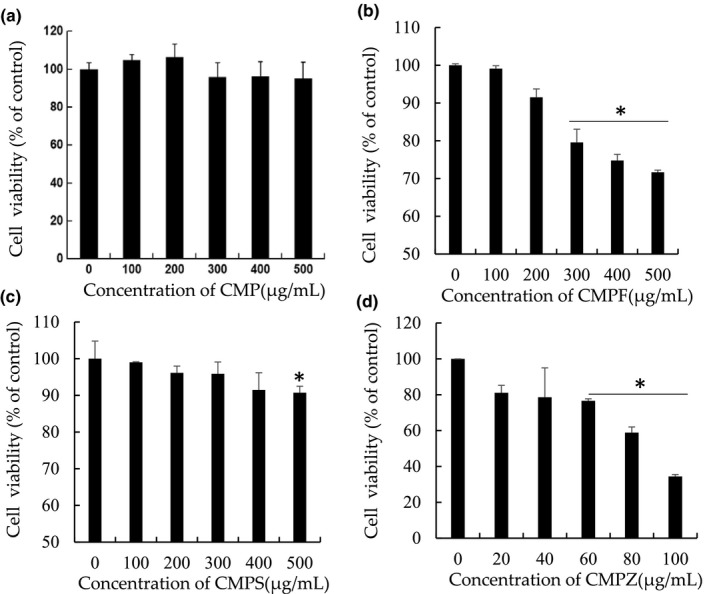
Effects of CMP, CMPF, CMPS, and CMPZ on cell viability. (a) MTT assay for CMP. (b) MTT assay for CMPF. (c) MTT assay for CMPS. (d) MTT assay for CMPZ. * *P* < .05

The inhibition of CMP, CMPF, CMPS, and CMPZ on the ovarian cancer cell line A2780 was studied using the CCK8 assay. An inhibitory effect of CMP was observed in the A2780 cell line. As shown in Figure 6a, the inhibition of A2780 cells by CMP in the concentration range of 100–500 μg/ml was very low, and the effect was not significant. CMPF inhibited the proliferation of A2780 cells, especially in the range of 300–500 μg/ml. Also, the proliferation of A2780 cells was inhibited by CMPS. At the concentration of 500 μg/ml, cell viability was 90.7% of that of the control group. CMPZ had the strongest inhibitory effect on the proliferation of A2780 cells. At the concentration of 20 μg/ml, the cell viability was 81.087% of that of the control group. It can be seen that, after chemical modification of the polysaccharide, the inhibition of proliferation of ovarian cancer cells was significantly enhanced, especially by the polysaccharide zinc.

Zinc is a necessary trace element for the human body. It is the second most abundant element in the human body and is mainly distributed in skin, bone, and muscle. It participates in the synthesis of a series of enzymes in the human body and plays a very important role in the growth, development, and immunity of the human body (Depciuch et al., 2017). Dong et al. (2018) found that the polysaccharide zinc of arteria rotunda had good in vitro antioxidant activity for 1,1‐diphenyl‐2‐picrylhydrazyl, hydroxyl radicals, and superoxide anions (Huang et al., 2015). Zhao et al. (2016) found that the polysaccharide zinc of *Lentinus edodes* had not only good antioxidant activity but also a good inhibitory effect on L929 tumor cells. Polysaccharide zinc has potential for further research studies and applications.

In recent years, there have been many studies on the antitumor activities of selenium, and there are great expectations for the application prospect of the selenium industry. Selenium mainly exists in four forms: Se(0), ${\text{SeO}}_{2}^{‐}$, Se^2−^, and elemental selenium. Selenium is an important component of many biological enzymes in the human body and has many biological functions, such as anticancer and detoxification. It is an important nutritional element in the human body. It has been shown that sodium selenite, selenomethylselenocysteine, and methylselenic acid have obvious antitumor effects, via cell oxidation and the induction of apoptosis. However, inorganic selenium represented by sodium selenite can cause poisoning. With the development of polysaccharides in recent years, the organic selenium form of polysaccharide selenate has shown advantages in terms of its antitumor effect of organic selenium and low toxicity. Deng found that selenium carrageenan had a good inhibitory effect on HepG2, MCF7, SGC7901, A549, and BEL7402 cancer cells. It is a promising area of research to develop new polysaccharide selenium materials with good effects.

### 3.5 CMPF, CMPS, and CMPZ increased levels of ROS

**FIGURE 7:**
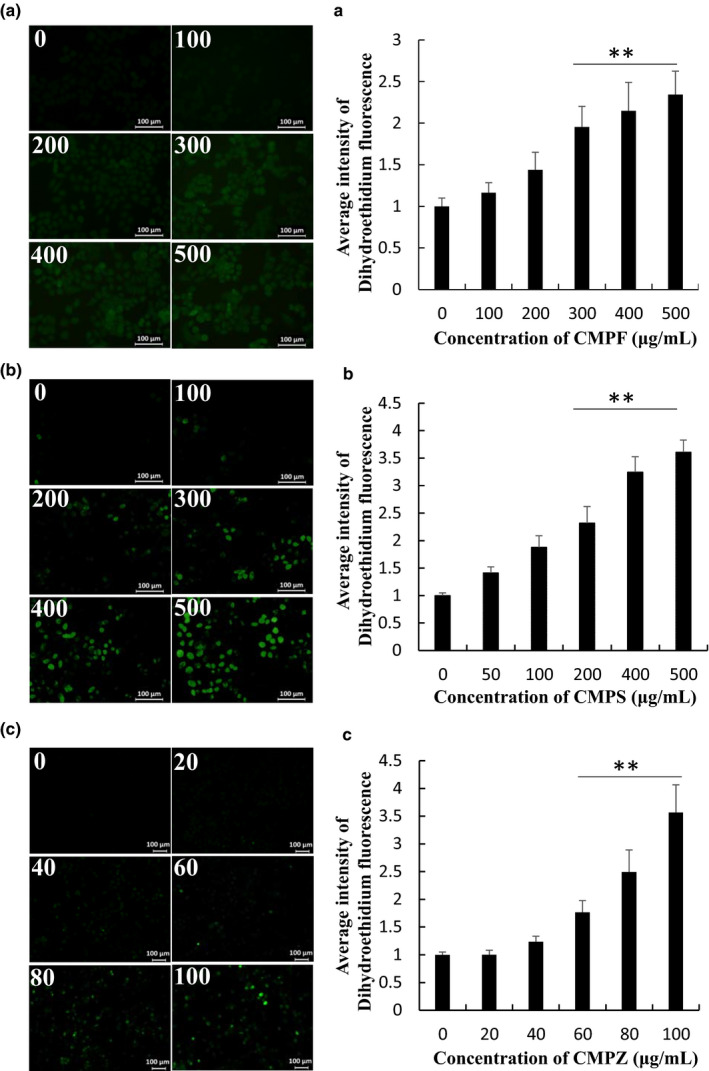
Effects of CMPF, CMPS, and CMPZ on ROS level in A2780 cells. (A) ROS assay for CMPF. (B) ROS assay for CMPS. (C) ROS assay for CMPZ. ** *P* < .01

ROS are active oxygen‐containing compounds produced in the process of aerobic metabolism. Medium to high concentrations of ROS can damage the dynamic balance of cells, cause oxidative stress, induce apoptosis, and even lead to necrosis of cells. As a signaling molecule, it can activate transcription factors through concentration regulation, and participate in many physiological processes such as cell proliferation, differentiation, and apoptosis. As shown in Figure 7, with the increase in the concentration of CMPF, CMPS, and CMPZ, ROS levels were increased, respectively, which indicates that CMPF, CMPS, and CMPZ had induced active oxygen in A2780 cells in a concentration‐dependent manner.

### 3.6 CMPF, CMPS, and CMPZ induced apoptosis

**FIGURE 8:**
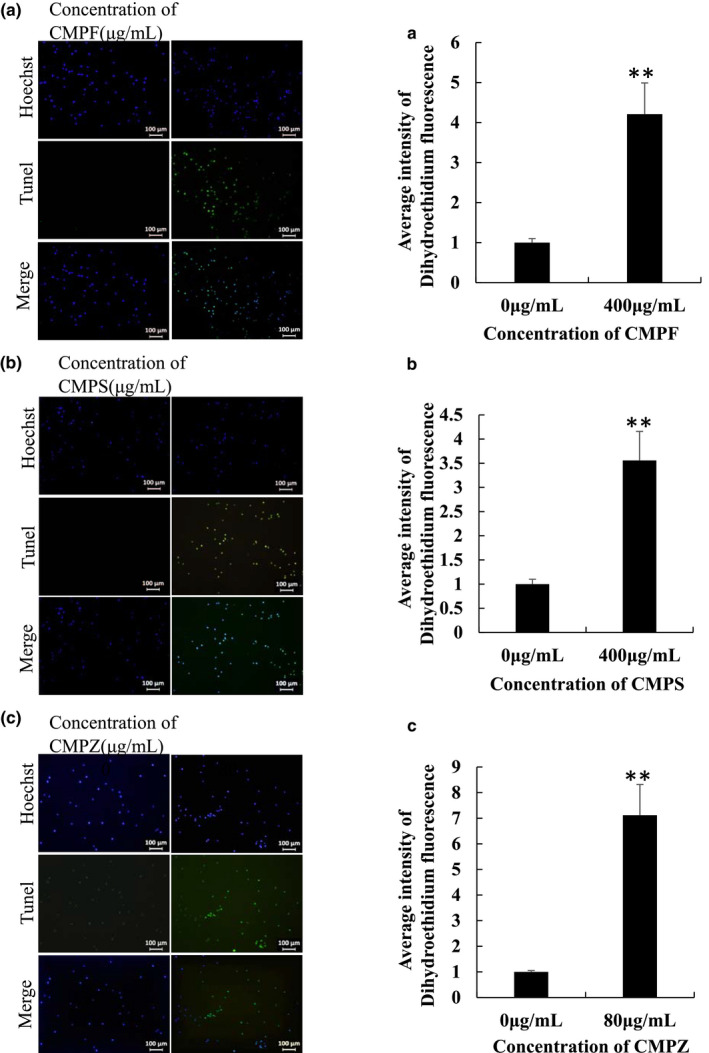
Effects of CMPF, CMPS, and CMPZ on apoptosis in A2780 cells. (A) TUNEL assay for CMPF. (B) TUNEL assay for CMPS. (C) TUNEL assay for CMPZ. ** P < .01

In the late stage of apoptosis, a large number of sticky 3‐OH terminals are produced by double or single‐strand breaks of chromosomal DNA. Under the effect of terminal deoxyribonucleotide transfer, fluorescein/enzyme‐labeled dUTP is combined to the 3‐terminal of DNA so that cell death can be detected by fluorescence. This method is called TUNEL. A2780 cells were treated, respectively, with CMPF and CMPS at the concentration of 400 and 80 μg/ml CMPZ for TUNEL detection. As shown in Figure 8, compared with the control group, the fluorescence intensity of the cells in the treatment group increased significantly. CMPF, CMPS, and CMPZ induced apoptosis in A2780 cells. It has been reported that CMP and its modified substances have been used in antitumor research.

CMP has a direct cytotoxic effect on mouse Ehrlich ascites cancer cells, as it inhibits DNA synthesis in the cell; this inhibition is dose‐dependent (Lin et al., 2004). Sulfated CMP can up‐regulate the expression of Bax and Fas, decrease the expression of Bcl‐2, and induce apoptosis in tumor cells (Meng et al., 2007; Yang et al., 2005). Polysaccharides are good chelating agents for metal ions because of their good structure and functional activity. The polysaccharide iron (III) complex promotes the proliferation of lymphocytes and enhances the activity of macrophages in mice, as well could also be used as an iron enhancer to assist in the treatment of iron deficiency anemia (Cui et al., 2017; Gao et al., 2019; Liu, Yin, et al., 2019; Liu, Liu, et al., 2019). Polysaccharide selenium can be used as a potential antioxidant, selenium supplement, and immune enhancer in the treatment of cancer and diabetes (Li et al., 2015; Pang & Chin, 2019; Wang et al., 2018; Yang & Zhang, 2016). Polysaccharide zinc could promote the apoptosis of cancer cells by activating the expression of caspase‐3, caspase‐8, and caspase‐9, inducing chromatin condensation, mitochondrial dysfunction, and excessive production of ROS. Therefore, polysaccharide zinc can be used as a candidate drug for tumor treatment and prevention (Liao et al., 2015). At the same time, polysaccharide zinc can also be used as a zinc supplement with a hypoglycemic effect (Zhang, Zhu, et al., 2019; Zhang, Khan, et al., 2019).

## 4 CONCLUSION

In this study, CMP was synthesized by a one‐step method with *P. cocos* as the research object, and the preparation process of CMPF, CMPS, and CMPZ was optimized by RSD. The characterization of CMPF, CMPS, and CMPZ was carried out by FTIR and NMR analysis; then, the antioxidant and antitumor activities of CMP together with its chemical modifications were studied. CMP did not contain nucleic acid or protein, and its carboxymethylated substitution degree was 0.6352. We determined that the optimal conditions for the synthesis of CMPF were a reaction pH of 7.45, reaction temperature of 74.29℃, and a ratio of trisodium citrate: polysaccharide, 0.64. The optimal conditions for the synthesis of CMPS were a mass ratio of sodium selenite to polysaccharide, 0.98, a volume fraction of nitric acid, 0.6%, and a reaction temperature of 69.2℃. The optimal conditions for the synthesis of CMPZ were a reaction time of 1.95 hr, pH of 7, and a reaction temperature of 67.79℃. CMP and its chemical modifiers had antioxidant capacities. As a comparison, CMPF and CMPZ improved the scavenging capacity of superoxide anion but little change with other capacities, and the antioxidant activity of CMPS was basically unchanged. CMP had no inhibitory effect on the proliferation of A2780 cells; on the contrary, CMPF, CMPS, and CMPZ had an obvious inhibitory effect. Compared with CMPF and CMPS, CMPZ could inhibit the proliferation of A2780 cells at a relatively low concentration (20–100 μg/ml) with a relatively short time (6 hr), produced a large amount of ROS in cells, and induced apoptosis at a concentration of 80 μg/ml.

## CONFLICT OF INTEREST

The authors declare no conflict of interest.

## AUTHOR CONTRIBUTIONS

Zhang XF came up with the idea and participated in writing the manuscript. Zhang XF, Yan YY, Yuan S, and Ma HH performed all literature surveys. All authors read and approved the final manuscript.

## ETHICAL APPROVAL

Ethics approval was not required for this research.

## Supporting information

Datas S1–S3

## Data Availability

Research data are not shared.
