# Supplementary material for: Structural modification and biological activities of carboxymethyl Pachymaran
Source: Food Sci Nutr. 2021 Jun 30;9(8):4335–48. doi: 10.1002/fsn3.2404 (PMC8358349; doi:10.1002/fsn3.2404)
Supplement: Supplementary file 1 — Datas S1–S3 [file FSN3-9-4335-s001.docx]

**Supplemental data 1**

**Optimization of Preparation Technology of CMPF**

According to Box-Benhnken design(BBD), combined with the single factor experimental results, three factors (pH, temperature (℃) and the ratio of trisodium citrate to polysaccharide) and three level designs were carried out. Response surface design (RSD) experiment was carried out with the iron content as the response value, and the experimental data were analysed with the design expert 8.0.6.1 software, and the equation was fitted to determine the best scheme of synthesize. **1 Single factor experiment** In this study, pH (X_1_, 6, 7, 8, 9 and 10), temperature (X_2_, 50, 60, 70, 80 and 90℃) and ratio of trisodium citrate to CMP (X_3_, 0.25, 0.50, 0.75, 1.00 and 1.25) were selected as the three variable factors to consider in the optimization of CMPF synthesis. The single-factor investigation was conducted using the control variable method with iron content as the response value. And the iron content was determined by phenanthroline spectrophotometry (Wang Chun-Yan, Wan Ji-Hong, Guo Hui-Min.(2002). Improvement in spectrometry to determine total Fe content. *Heilongjiang Enviromental Jouknal. 26*(1): 73-74.).

- 1. **Effect of pH on synthesis of CMPF**

The controlled variable method was used to control the temperature (60℃) and the ratio of trisodium citrate to CMP (0.5). The pH (6, 7, 8, 9 and 10) was only changed to investigate the effect of pH on iron content of CMPF. The result was shown in **Figure S1-1**. The iron content of CMPF increased as pH increased from 6.0 to 8.0, reaching maximum iron content at pH 8, and then decreased.


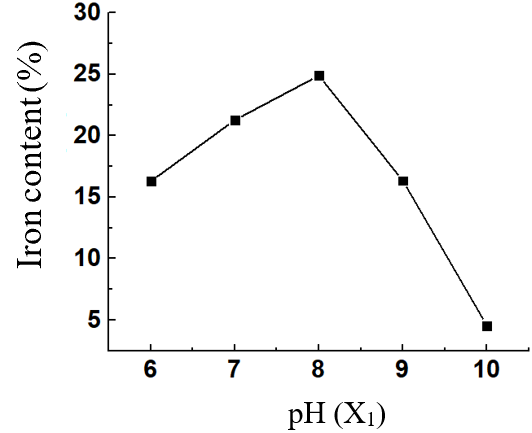


**Figure S1-1. Effect of pH on synthesis of CMPF**

- 1. **Effect of temperature on synthesis of CMPF**

At a fixed the ration of pH and temperature of 8 and 60℃, respectively. Only the temperature (50, 60, 70, 80 and 90℃) was changed to investigate the effect of temperature on iron content of CMPF. The result was shown in **Figure S1-2**. The iron content of CMPF increased as the temperature was increases from 50℃ to 70℃, the peak of iron content was achieved (24%) at 70℃, and then the iron content of CMPF began to decline as the temperature was further increased.


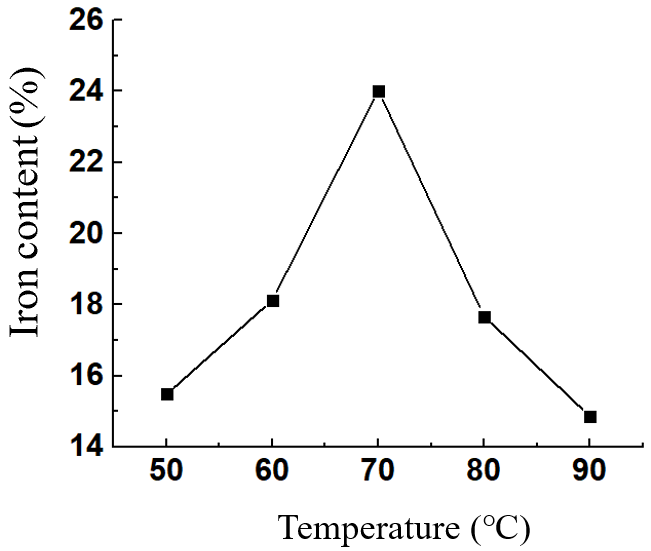


**Figure S1-2. Effect of temperature on synthesis of CMPF**

- 1. **Effect of the ratio of trisodium citrate and CMP on iron content of CMPF**

At a fixed pH and temperature of 8.0 and 70℃, respectively. Only the ratio of trisodium citrate and CMP (0.25, 0.50, 0.75, 1.00 and 1.25) was changed. The results are shown in **Figure S1-3**. The iron content of CMPF increased as the ratio of trisodium citrate and CMP was increases from 0.25 to 0.75, the peak of iron content was achieved (21%) at 0.75, and then the iron content of CMPF began to decline.


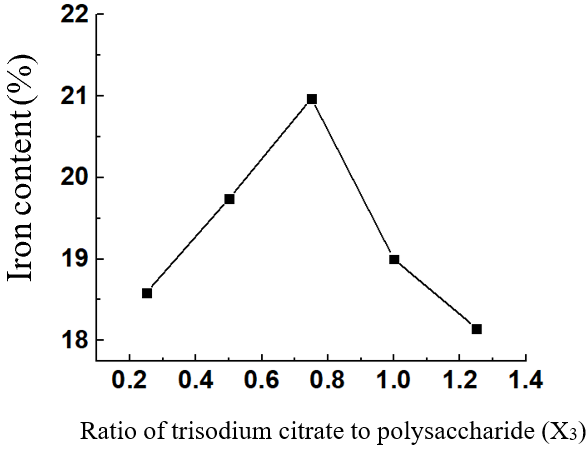


**Figure S1-2. Effect of the ratio of trisodium citrate to CMP on synthesis of CMPF**

1. **Response surface optimization experiment**

According to the Box-Benhnken design (BBD) principle, a three-factor three-level design was performed in combination with the results of the single-factor experiment. Response surface methodology (RSM) was applied to investigate the effect of three variables on iron content of CMPF. The design data were analyzed using Design-Expert 8.0.6.1 software to fit the equation.

**Table S1-1**

Levels and code of variables used in Box-Behnken design

| Variable | Symbols  Coded | | Coded levels  -1 0 1 | | |
| --- | --- | --- | --- | --- | --- |
| pH | X_1_ | | 7 | 8 | 9 |
| Exaction temperature (℃) | | X_2_ | 60 | 70 | 80 |
| Ration of Trisodium citrate: CMP | X_3_ | | 0.5 | 0.75 | 1 |

- 1. **Response Surface Optimization Experiment Design**

Based on the results of the single-factor experiment, the subsequent three-factor three-level Box-behnken experimental scheme was designed. The range of independent variables and the corresponding response values were shown in **Table S1-1**. A 17-run BBD was applied to statistically optimize the iron content of CMPF (**Table S1-2**).

- 1. **Response surface analysis**

Based on the BBD design, the results of 17 sets of response surface experiments and the experimental values were presented in **Table S1-2**. Using the iron content as the response value, based on the experimental data obtained from the multiple regression analysis method, the predicted response Y for the iron content of CMPF can be fitted into the following second-order polynomial equation:

Y=-486.23+99.053X_1_+3.90758X_2_+17.097X_3_-0.073X_1_X_2_+5.28X_1_X_3_-0.011X_2_X_3_-6.51175 X_1_^2^-0.022592 X_2_^2^-43.42800 X_3_^2^
 where Y is the iron content of CMPF (%), and X_1_, X_2_ and X_3_ are the coded values of the tested pH, temperature and the ratio of trisodium citrate to CMP, respectively. The analysis of variance (ANOVA) results of the response surface quadratic model are summarized in **Table S1-3**. According to **Table S1-3**, the model established in this experiment (P <0.001) is extremely significant, and the mismatch term (P = 0.4440> 0.05) is not significant. These data indicate that the response surface model established in this experiment was feasible for the optimal preparation of CMPF. The signal-to-noise ratio Adeq Precisior = 48.782 was relatively high. The model can predict the experimental results, and the model correction judgment coefficient R^2^_Adj_ = 0.9953, indicating that the model can prove the predicted 99.53% response value. Judgment coefficient R^2^ = 0.9980, indicating that the model fits well, and the model can be used to analyze and predict the iron content of CMPF.

**Table S1-2**

Box-Behnken experimental design and the results for extraction yield of polysaccharides

| **Run** | **X_1_** | **X_2_** | **X_3_** | **Fe (%)** |
| --- | --- | --- | --- | --- |
| 1 | -1 | 1 | 0 | 30.91 |
| 2 | 1 | 0 | 1 | 15.19 |
| 3 | -1 | 0 | -1 | 30.77 |
| 4 | 0 | 0 | 0 | 30.93 |
| 5 | 1 | 1 | 0 | 16.39 |
| 6 | 0 | 0 | 0 | 30.81 |
| 7 | 0 | -1 | 1 | 22.89 |
| 8 | 0 | 0 | 0 | 30.55 |
| 9 | -1 | -1 | 0 | 26.38 |
| 10 | 0 | 1 | -1 | 28.99 |
| 11 | 0 | 1 | 1 | 25.86 |
| 12 | -1 | 0 | 1 | 24.63 |
| 13 | 1 | 0 | -1 | 16.05 |
| 14 | 0 | -1 | -1 | 25.61 |
| 15 | 0 | 0 | 1 | 31.57 |
| 16 | 1 | -1 | 1 | 14.78 |
| 17 | 0 | 0 | 1 | 30.57 |

**Table S1-3**

Analysis of variance of the experimental results of the BBD

| **Varibles** | **Sun of squares** | **df** | **Mean square** | **F-value** | **p-Value**  **Prob.>F** | |
| --- | --- | --- | --- | --- | --- | --- |
| Model | 615.84 | 9 | 68.43 | 380.19 | | < 0.0001** |
| X_1_ | 316.01 | 1 | 316.01 | 5.63 | | < 0.0001** |
| X_2_ | 18.57 | 1 | 18.57 | 3.82 | | < 0.0001** |
| X_3_ | 21.62 | 1 | 21.62 | 12.17 | | < 0.0001** |
| X_1_X_2_ | 2.13 | 1 | 2.13 | *11.84* | | 0.0108 |
| X_1_X_3_ | 6.97 | 1 | 6.97 | 38.72 | | 0.0004 |
| X_2_X_3_ | 3.025E-003 | 1 | 3.025E-003 | 0.017 | | 0.9005 |
| X_1_^2^ | 178.54 | 1 | 178.54 | 992.00 | | < 0.0001** |
| X_2_^2^ | 21.49 | 1 | 21.49 | 119.41 | | < 0.0001** |
| X_3_^2^ | 31.02 | 1 | 31.02 | 172.35 | | < 0.0001** |
| Pesidual | 1.26 | 7 | 0.18 | - | | - |
| Lack of fit | 0.57 | 3 | 0.19 | 1.11 | | 0.4440 |
| Pure error | 0.69 | 4 | 0.17 | - | | - |
| Correlation total | 617.10 | 16 | - | - | | - |
| R^2^=0.9980 | R^2^_Adj_=0.9953 | R^2^_pred_=0.9834 Adeq Precisior=48.782 | | | | |

**Means significant differences (P<0.01), *Means significant differences (P< 0.05).

As shown in **Figure S1-4** to **S1-6**, 3D response surfaces and 2D contour plots were generated, which shown the interaction of the variables and the optimal level of each variable for maximum response.


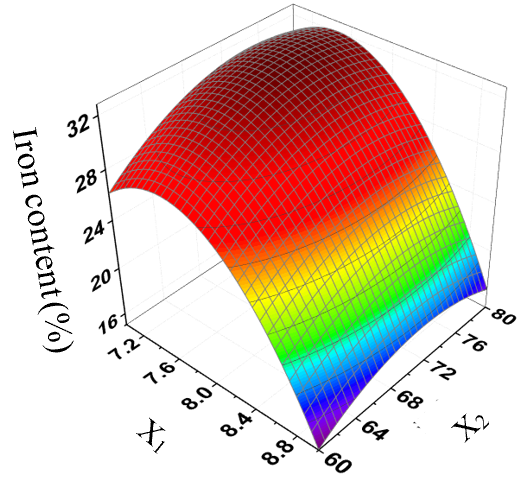


X_1_

X_2_


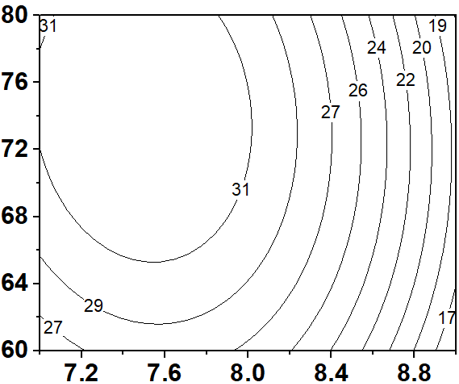


pH

**Figure S1-4** The response surface plots of the effect of ultrafiltration pH(X_1_), temperature(X_2_) and their reciprocal interaction on the iron content of CMPF (%).


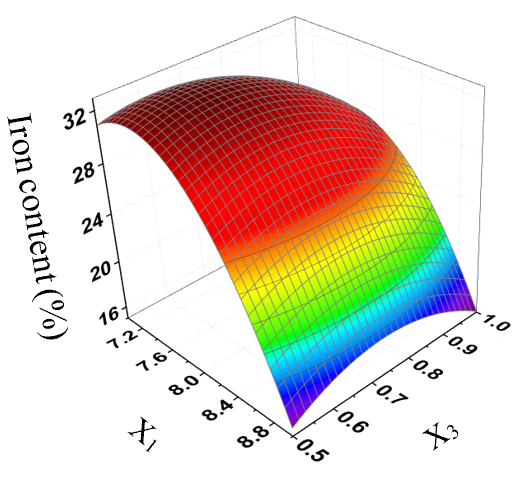


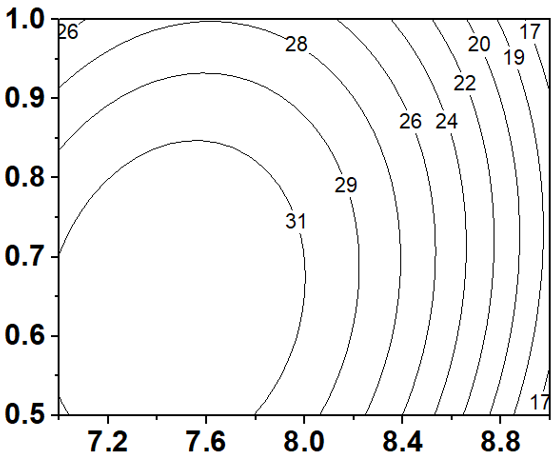


X_1_

X_3_

**Figure S1-5** The response surface plots of the effect of ultrafiltration pH(X_1_), ration of trisodium citrate: CMP (X_3_) and their reciprocal interaction on the iron content of CMPF (%).


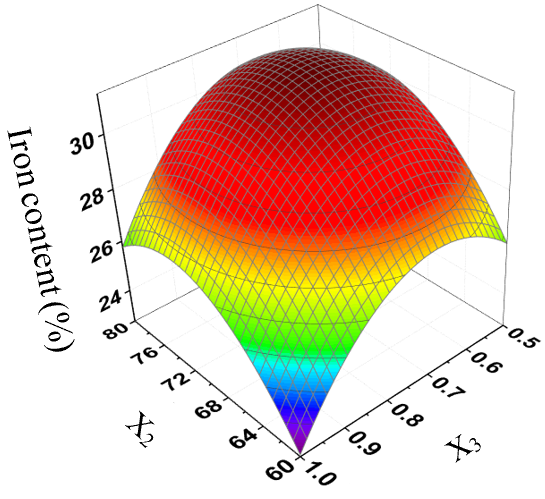


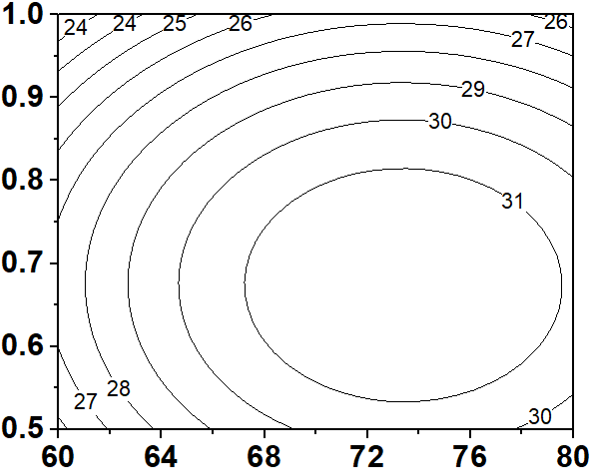


X_2_

X_3_

**Figure S1-6** The response surface plots of the effect of temperature(X_2_), ration of trisodium citrate: CMP (X_3_) and their reciprocal interaction on the iron content of CMPF (%).

**Figure S1-4** show the interaction effect of pH(X_1_) and temperature(X_2_) on the iron content of CMPF when ration of trisodium citrate: CMP (X_3_) was constant. The iron content increased as the pH and temperature increased, but decreased when the pH and temperature ascended past a certain point. From the contours of the graphics and the density degree, the interaction of pH and temperature was significant. As presented in **Figure S1-5**, the response surface plots of the effect of ultrafiltration pH(X_1_), ration of trisodium citrate: CMP (X_3_) and their reciprocal interaction on the iron content of CMPF (%). The effect surface of the reaction X_1_ was relatively steeper, the contour lines are relatively denser, and the reaction of X_3_ was relatively smooth, and the contours were relatively sparse. As shown in **Figure S1-5**, the temperature (X_2_) of the reaction is relatively steeper, the contours are relatively dense, and the reaction of X_3_ is relatively smooth, and the contours were relatively sparse, so the reaction temperature was more significant than X_3_.

**2.5 Validation of polysaccharide iron preparation optimization process**

According to these results, the optimal synthesis process conditions predicted by the response surface software were as follows: a pH of 7.45, the reaction temperature of 74.29 ° C and the ratio of the trisodium citrate: polysaccharide of 0.64. Under these optimal conditions, and the experimental iron content of CMPF was 31.91%. Compared with the predicted value of 33.31%, there was a 95.8% agreement, and the deviation rate was 4.2%, indicating that the response surface model is good and can be used to predict the iron content of CMPF.

**Supplemental data 2**

### Optimization of Preparation Technology of CMPS

### According to the BBD, combined with the single factor experimental results, three factors (the ratio of sodium selenite to polysaccharide, volume fraction of nitric acid (%) and temperature) and three level designs were carried out. RSD experiment was carried out with the selenium content as the response value, and the experimental data were analysed with the design expert 8.0.6.1 software, and the equation was fitted to determine the best scheme of synthesize. 1 Single factor experiment In this study, the ratio of sodium selenite to polysaccharide (X_1_, 0.6, 0.8, 1.0, 1.2 and 1.4), volume fraction of nitric acid (X_2_, 0.2, 0.4, 0.6, 0.8 and 1%) and temperature (X_3_, 50, 60, 70, 80 and 90℃) were selected as the three variable factors to consider in the optimization of CMPS synthesis. The single-factor investigation was conducted using the control variable method with selenium content as the response value. And the selenium content was determined by o-phenylenediamine method (Gao Yi-Xia, Yuan Yi-Jun, Zhou Xiang-Jun, Wang Feng-Xia, Zhang Ji. (2012). [Preparation and characterization of polysaccharide selenate of tamarind seeds](http://www.cqvip.com/QK/95650X/201207/42673027.html). *Chinese Journal of Pharmaceutical Analysis. 32*(7): 1222-1226.).

**1.1 Effect of Ration of Sodium selenite: CMP on Synthesis of CMPS**

The controlled variable method was used to control the volume fraction of nitric acid (0.2%) and temperature (60℃). The ration of sodium selenite to CMP (0.6, 0.8, 1.0, 1.2 and 1.4) was only changed to investigate the effect on selenium content of CMPS. The result was shown in **Figure S2-1**. The selenium content of CMPS increased as the ration of sodium selenite to CMP increased from 0.6 to 1.0, reaching maximum selenium content at 1.0, and then decreased.


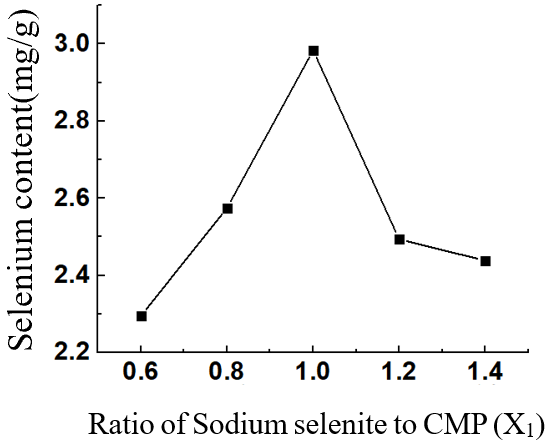


**Figure S2-1. Effect of the ration of sodium selenite to CMP on synthesis of CMPS**

**1.2 Effect of volume fraction of Nitric acid on synthesis of CMPS**

At a fixed ration of sodium selenite to CMP and temperature of 1.0 and 60℃, respectively. Only the volume fraction of nitric acid (X_2_, 0.2, 0.4, 0.6, 0.8 and 1%) was changed. The results was shown in **Figure S2-2**. The selenium content of CMPS increased as the volume fraction of nitric acid was increases from 0.2% to 0.6%, the peak of selenium content was achieved (3.1mg/g) at 0.6%, and then the selenium content of CMPS began to decline.


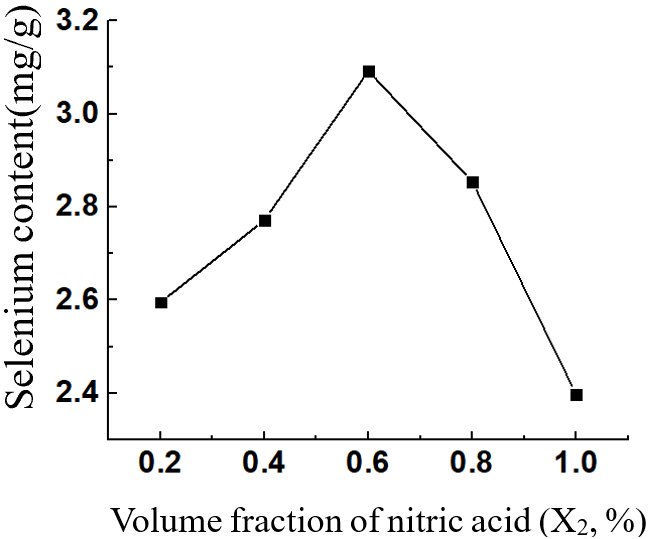


**Figure S2-2. Effect of volume fraction of nitric acid on synthesis of CMPS**

**1.3 Effect of temperature on synthesis of CMPS**

At a fixed the ration of sodium selenite to CMP and volume fraction of nitric acid of 1.0 and 0.6%, respectively. Variation in temperature (50, 60, 70, 80 and 90℃) also altered the selenium content of CMPS. The result was shown in **Figure S3-3**. Only the temperature was changed to investigate the effect of temperature on selenium content of CMPS. The selenium content of CMPS increased as the temperature was increased from 50℃ to 70℃, the peak of selenium content was achieved (3.08mg/g) at 70℃, and then the selenium content of CMPS began to decline as the temperature was further increased.


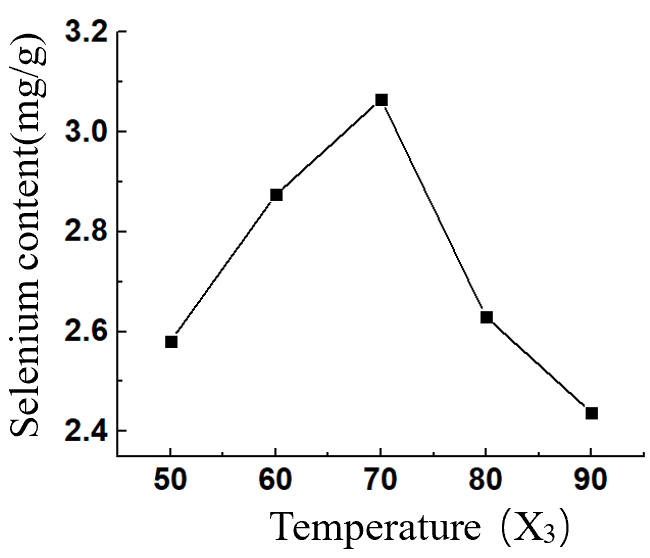


**Figure S2-3. Effect of temperature on synthesis of CMPS**

1. **Response surface optimization experiment**

According to the Box-Benhnken design (BBD) principle, a three-factor three-level design was performed in combination with the results of the single-factor experiment. Response surface methodology (RSM) was applied to investigate the effect of three variables on selenium content of CMPS. The design data were analyzed using Design-Expert 8.0.6.1 software to fit the equation.

- 1. **Response Surface Optimization Experiment Design**

Based on the results of the single-factor experiment, the subsequent three-factor three-level Box-behnken experimental scheme was designed. The range of independent variables and the corresponding response values were shown in **Table S2-1**. A 17-run BBD was applied to statistically optimize the selenium content of CMPS (**Table S2-2**).

**Table S3-1**

Levels and code of variables used in Box-Behnken design

| **Variable** | **Symbols**  **Coded** | **Coded levels**  **-1 0 1** | | |
| --- | --- | --- | --- | --- |
| **Ration of Sodium selenite: CMP** | X_1_ | 0.8 | 1 | 1.2 |
| **volume fraction of nitric acid (%)** | X_2_ | 0.4 | 0.6 | 0.8 |
| **Exaction temperature(℃)** | X_3_ | 60 | 70 | 80 |

- 1. **Response surface analysis**

Based on the BBD design, the results of 17 sets of response surface experiments and the experimental values were presented in **Table S2-2**. Using the selenium content as the response value, based on the experimental data obtained from the multiple regression analysis method, the predicted response Y for the selenium content of CMPS can be fitted into the following second-order polynomial equation:

Y=-27.49475+ 12.61937X_1_+ 12.43437X_2_+ 0.59478X_3_+1.15X_1_X_2_-0.011125X_1_X_3_ + 0.000125X_2_X_3_- 6.41875 X_1_^2^- 11.23125 X_2_^2^- 0.00422X_3_^2^

where Y is the selenium content of CMPS (mg/g), and X_1_, X_2_ and X_3_ are the coded values of the tested the ration of sodium selenite to CMP, volume fraction of nitric acid (%) and temperature (℃), respectively. The analysis of variance (ANOVA) results of the response surface quadratic model are summarized in **Table S2-3**. According to **Table S2-3**, the model established in this experiment (P <0.001) was extremely significant, and the mismatch term (P = 0.4400> 0.05) was not significant. These data indicate that the response surface model established in this experiment was feasible for the optimal preparation of CMPS. The signal-to-noise ratio Adeq Precisior = 30.343 was relatively high. The model can predict the experimental results, and the model correction judgment coefficient R^2^_Adj_=0.9874, indicating that the model can prove the predicted 98.74% response value. Judgment coefficient R^2^=0.9945, indicating that the model fits well, and the model can be used to analyze and predict the selenium content of CMPS.

**Table S2-2**

Box-Behnken experimental design and the results for extraction yield of polysaccharides

| **Run** | **X_1_** | **X_2_** | **X_3_** | **Se(mg/g)** |
| --- | --- | --- | --- | --- |
| 1 | 1 | 1 | 0 | 2.329 |
| 2 | -1 | -1 | 0 | 2.343 |
| 3 | 1 | 0 | -1 | 2.316 |
| 4 | 0 | 0 | 0 | 3.038 |
| 5 | 0 | 0 | 0 | 2.950 |
| 6 | 0 | 0 | 0 | 3.025 |
| 7 | 1 | -1 | 0 | 2.172 |
| 8 | -1 | 1 | 0 | 2.316 |
| 9 | 0 | 0 | 0 | 2.956 |
| 10 | 1 | 0 | 1 | 2.152 |
| 11 | 0 | 1 | 1 | 2.057 |
| 12 | -1 | 0 | 1 | 2.363 |
| 13 | 0 | -1 | -1 | 2.193 |
| 14 | -1 | 0 | -1 | 2.438 |
| 15 | 0 | 1 | -1 | 2.220 |
| 16 | 0 | -1 | 1 | 2.029 |
| 17 | 0 | 0 | 0 | 3.011 |

**Table S2-3**

Analysis of variance of the experimental results of the BBD

| **Varibles** | **Sun of squares** | **df** | **Mean square** | **F-value** | **p-Value Prob.>F** |
| --- | --- | --- | --- | --- | --- |
| Model | 2.17 | 9 | 2.17 | 139.97 | < 0.0001** |
| X_1_ | 0.030 | 1 | 0.030 | 17.51 | < 0.0041** |
| X_2_ | 4.278E-003 | 1 | 4.278E-003 | 2.49 | 0.1589 |
| X_3_ | 0.040 | 1 | 0.040 | 23.27 | < 0.0019** |
| X_1_×X_2_ | 8.464E-003 | 1 | 8.464E-003 | 4.92 | 0.0621 |
| X_1_×X_3_ | 1.980E-003 | 1 | 1.980E-003 | 1.15 | 0.3190 |
| X_2_×X_3_ | 2.500E-007 | 1 | 2.500E-007 | 1.453E-004 | 0.9907 |
| X_1_^2^ | 0.28 | 1 | 0.28 | 161.30 | < 0.0001** |
| X_2_^2^ | 0.85 | 1 | 0.85 | 493.85 | < 0.0001** |
| X_3_^2^ | 0.75 | 1 | 0.75 | 435.76 | < 0.0001** |
| Pesidual | 0.012 | 7 | 1.721E-003 | - | - |
| Lack of fit | 5.499E-003 | 3 | 1.833E-003 | 1.12 | 0.4400 |
| Pure error | 6.546E-003 | 4 | 1.636E-003 | - | - |
| Correlation total | 2.18 | 16 | - | - | - |
| R^2^=0.9945 | R^2^_Adj_=0.9874 | R^2^_pred_=0.9549 Adeq Precisior=30.343 | | | |

**Means significant differences (P<0.01), *Means significant differences (P< 0.05).

As shown in **Figure S2-4** to **S2-6**, 3D response surfaces and 2D contour plots were generated, which shown the interaction of the variables and the optimal level of each variable for maximum response.


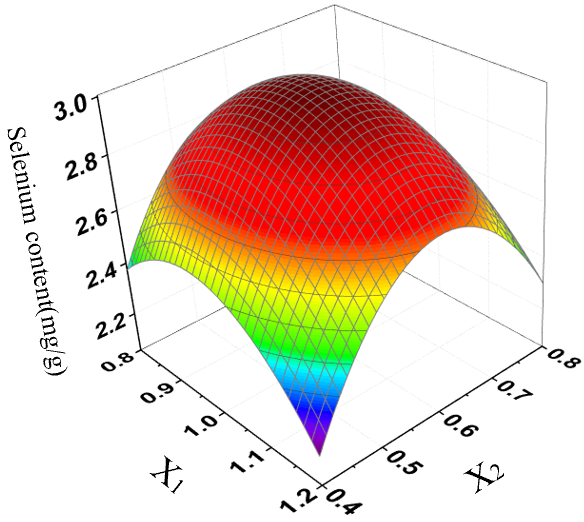


X_1_

X_2_


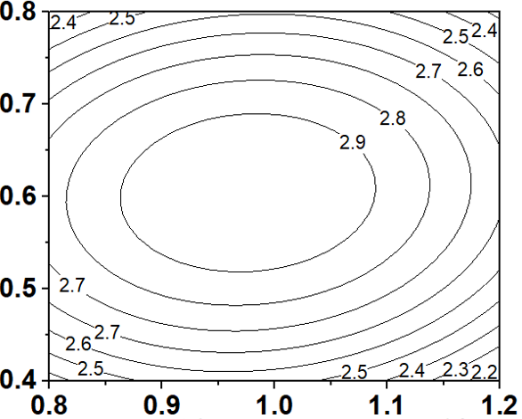


**Figure S2-4** The response surface plots of the effect of the ration of sodium selenite to CMP(X_1_), volume fraction of nitric acid (%, X_2_) and their reciprocal interaction on the selenium content of CMPS (mg/g).


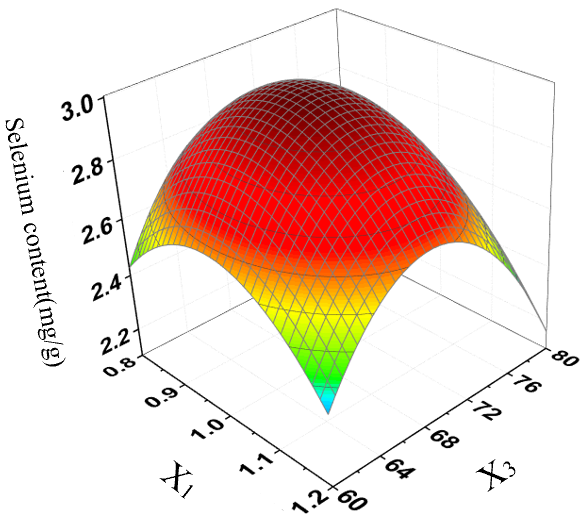


X_3_

X_1_


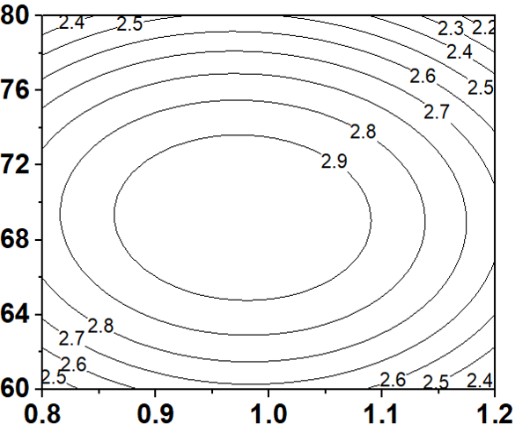


**Figure S2-5** The response surface plots of the effect of the ration of sodium selenite: CMP(X_1_), temperature(X_3_) and their reciprocal interaction on the selenium content of CMPS (mg/g).


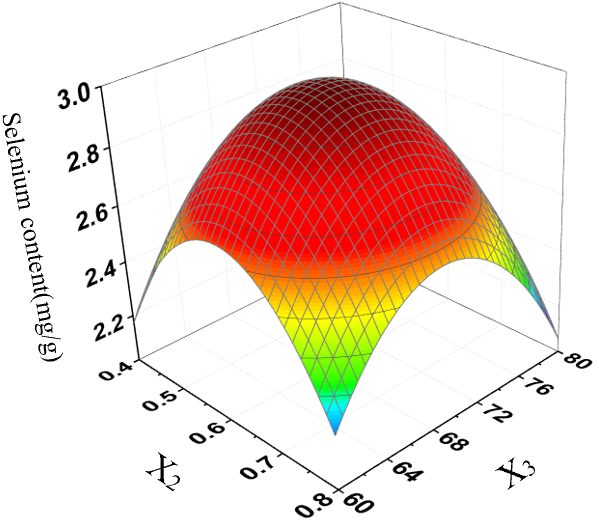


X_3_

X_2_


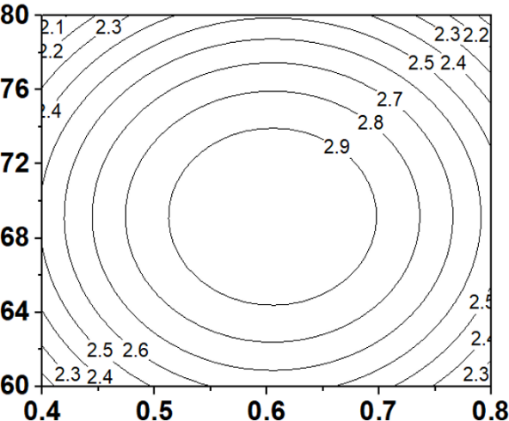


**Figure S2-6** The response surface plots of the effect of volume fraction of nitric acid (%, X_2_), temperature(X_3_) and their reciprocal interaction on the selenium content of CMPS (mg/g).

The interaction and influence of various factors in the response surface can be directly reflected by the contour density in the contour map and the steepness in the response surface map in **Figure S2-4** to **S2-6**. It can be seen from **Figure S2-4** to **S2-6** that there was a maximum value in the range of the highest point and contour line of the response surface, and the highest point in the figure was also the center point of the smallest ellipse in the contour line. As shown in the figure above, the contour map of CMPS shows that the ellipse shows that the interaction between various factors is very strong, and the effect on the synthesis of CMPS is significant.

**2.3 Validation of CMPS preparation optimization process**

According to these results, the optimal synthesis process conditions predicted by the response surface software were as follows: the ration of sodium selenite to CMP=0.98, volume fraction of nitric acid of 0.6% and temperature 69.2°C. Under these optimal conditions, and the experimental selenium content of CMPS was 3.067mg/g. Compared with the predicted value of 3.002mg/g, there was a 97.83% agreement, and the deviation rate was 2.17%, indicating that the response surface model is good and can be used to predict the iron content of CMPS.

**Supplemental data 3**

**Optimization of Preparation Technology of CMPZ**

According to the BBD and the results of single-factor experiments, three factors (time (h), pH, and temperature) and three level designs were carried out. RSM experiment was carried out with zinc content as the response value. The experimental data was analysed with design expert 8.0.6.1 software, and the equation was fitted to determine the best scheme of synthesize. **1 Single factor experiment** In this study, time(X_1_, 1, 2, 3, 4 and 5h), pH (X_2_, 4, 5, 6, 7 and 8) and temperature (X_3_, 40, 50, 60, 70 and 80℃) were selected as the three variable factors to consider in the optimization of CMPZ synthesis. The single-factor investigation was conducted using the control variable method with zinc content as the response value. And the zinc content was determined by phenanthroline spectrophotometry. The main method of zinc content was dithizone method (Yu Jing, Li Wen-Xiu, Wang Xue-Feng, Huang Xue-Li. (2014). Improvement of spectrophotometry method with dithizone for determination of zinc ions in aqueous. *Tian Jin Chemical. 28*(1):36-39.)

**1.1 Effect of time on Synthesis of CMPZ**

The controlled variable method was used to control the pH (7) and temperature (60℃). The time (1, 2, 3, 4 and 5h) was only changed to investigate the effect of time on zinc content of CMPZ. The result was shown in **Figure S3-1**. The zinc content of CMPZ increased as time increased from 1h to 5h, reaching maximum zinc content at 2h, and then decreased.


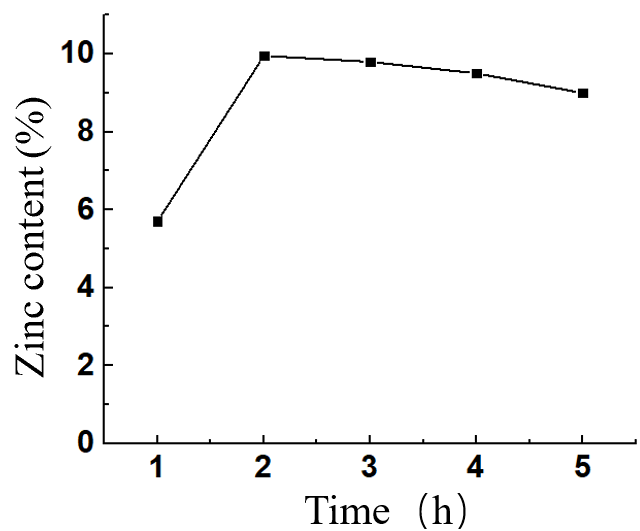


**Figure S3-1. Effect of time on synthesis of CMPZ**

**1.2 Effect of pH on synthesis of CMPZ**

At a fixed time and temperature of 2h and 60℃, respectively. Only the pH (4, 5, 6, 7 and 8) was changed. The results are shown in **Figure S3-2**. The zinc content of CMPZ increased as the pH was increases from 4 to 6, the peak of zinc content was achieved (11.4%) at pH=6, and then the zinc content of CMPZ began to decline.


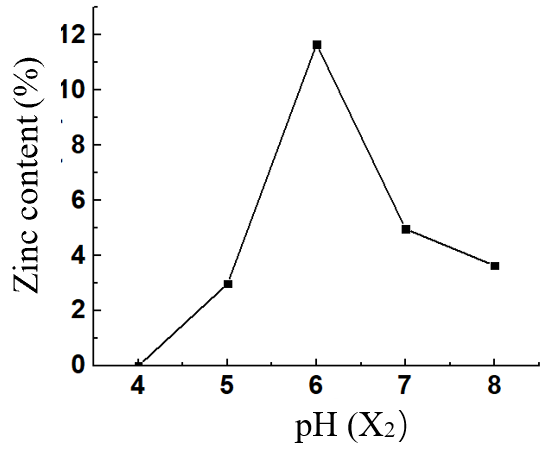


**Figure S3-2. Effect of pH on synthesis of CMPZ**

**1.3 Effect of temperature on synthesis of CMPZ**

At a fixed time and pH of 2h and 6, respectively. Variation in temperature (40, 50, 60, 70 and 80℃) also altered the zinc content of CMPZ. The result was shown in **Figure S3-3**. Only the temperature was changed to investigate the effect of temperature on zinc content of CMPZ. The zinc content of CMPZ increased as the temperature was increases from 40℃ to 70℃, the peak of zinc content was achieved (7.1%) at 70℃, and then the zinc content of CMPZ began to decline as the temperature was further increased.


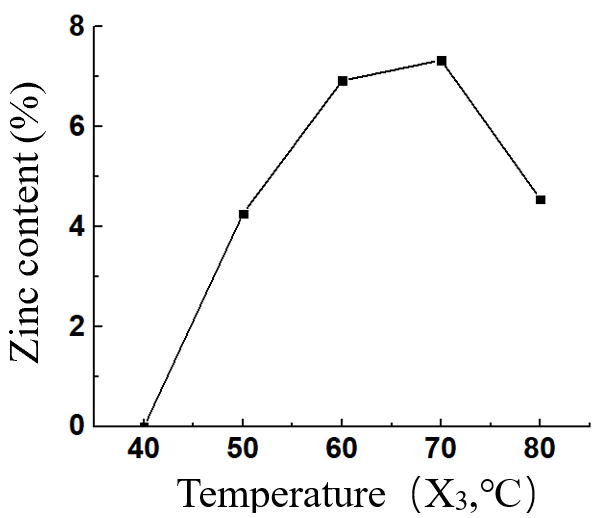


**Figure S3-3. Effect of temperature on synthesis of CMPZ**

1. **Response surface optimization experiment**

According to the Box-Benhnken design (BBD) principle, a three-factor three-level design was performed in combination with the results of the single-factor experiment. Response surface methodology (RSM) was applied to investigate the effect of three variables on zinc content of CMPZ. The design data were analyzed using Design-Expert 8.0.6.1 software to fit the equation.

**Table S3-1**

Levels and code of variables used in Box-Behnken design

| **Variable** | **Symbols**  **Coded** | | **Coded levels**  **-1 0 1** | | | |
| --- | --- | --- | --- | --- | --- | --- |
| **Time(h)** | | X_1_ | | 1 | 2 | 3 |
| **pH** | | X_2_ | | 5 | 6 | 7 |
| **Exaction temperature(℃)** | | X_3_ | | 60 | 70 | 80 |

- 1. **Response Surface Optimization Experiment Design**

Based on the results of the single-factor experiment, the subsequent three-factor three-level Box-behnken experimental scheme was designed. The range of independent variables and the corresponding response values were shown in **Table S3-1**. A 17-run BBD was applied to statistically optimize the zinc content of CMPZ (**Table S3-2**).

- 1. **Response surface analysis**

Based on the BBD design, the results of 17 sets of response surface experiments and the experimental values were presented in **Table S3-2**. Using the zinc content as the response value, based on the experimental data obtained from the multiple regression analysis method, the predicted response Y for the zinc content of CMPZ can be fitted into the following second-order polynomial equation:

Y=-130.6885+ 21.797X_1_+ 0.48975X_2_+ 3.33158X_3_- 0.965X_1_X_2_+ 0.065X_1_X_3_- 0.01975X_2_X_3_- 4.98675 X_1_^2^+0.31075 X_2_^2^- 0.024493X_3_^2^

where Y is the zinc content of CMPZ (%), and X_1_, X_2_ and X_3_ are the coded values of the tested time(h), pH and temperature (℃), respectively. The analysis of variance (ANOVA) results of the response surface quadratic model are summarized in **Table S3-3**. According to **Table S3-3**, the model established in this experiment (P <0.001) is extremely significant, and the mismatch term (P = 0.2937> 0.05) is not significant. These data indicate that the response surface model established in this experiment was feasible for the optimal preparation of CMPZ. The signal-to-noise ratio Adeq Precisior = 11.34 was relatively high. The model can predict the experimental results, and the model correction judgment coefficient R^2^_Adj_=0.8832, indicating that the model can prove the predicted 88.32% response value. Judgment coefficient R^2^=0.9485, indicating that the model fits well, and the model can be used to analyze and predict the zinc content of CMPZ.

**Table S3-2**

Box-Behnken experimental design and the results for extraction yield of polysaccharides

| **Run** | **X_1_** | **X_2_** | **X_3_** | **Zn (%)** |
| --- | --- | --- | --- | --- |
| 1 | 1 | 0 | 1 | 3.43 |
| 2 | 0 | 0 | 0 | 9.25 |
| 3 | 0 | -1 | -1 | 8.00 |
| 4 | 0 | 0 | 0 | 9.75 |
| 5 | -1 | -1 | 0 | 2.43 |
| 6 | 1 | 1 | 0 | 5.30 |
| 7 | 0 | 1 | 1 | 6.34 |
| 8 | 1 | -1 | 0 | 4.67 |
| 9 | 0 | -1 | 1 | 5.67 |
| 10 | 0 | 0 | 0 | 8.08 |
| 11 | -1 | 0 | 1 | 0.00 |
| 12 | -1 | 0 | -1 | 2.01 |
| 13 | -1 | 1 | 0 | 6.92 |
| 14 | 0 | 0 | 0 | 9.75 |
| 15 | 0 | 1 | -1 | 9.46 |
| 16 | 0 | 0 | 0 | 10.70 |
| 17 | 1 | 0 | -1 | 2.84 |

**Table S3-3**

Analysis of variance of the experimental results of the BBD

| **Varibles** | **Sun of squares** | **df** | **Mean square** | **F-value** | **p-Value Prob.>F** |
| --- | --- | --- | --- | --- | --- |
| Model | 156.82 | 9 | 17.42 | 14.44 | 0.0010** |
| X_1_ | 2.98 | 1 | 2.98 | 2.47 | 0.1603 |
| X_2_ | 6.57 | 1 | 6.57 | 5.44 | 0.0524 |
| X_3_ | 5.90 | 1 | 5.90 | 4.89 | 0.0627 |
| X_1_X_2_ | 3.72 | 1 | 3.72 | 3.09 | 0.1224 |
| X_1_X_3_ | 1.69 | 1 | 1.69 | 1.40 | 0.2753 |
| X_2_X_3_ | 0.16 | 1 | 0.16 | 0.13 | 0.7298 |
| X_1_^2^ | 104.71 | 1 | 104.71 | 86.76 | < 0.0001** |
| X_2_^2^ | 0.41 | 1 | 0.41 | 0.34 | 0.5798 |
| X_3_^2^ | 25.26 | 1 | 25.26 | 20.93 | 0.0026** |
| Pesidual | 8.45 | 7 | 1.21 | - | - |
| Lack of fit | 4.80 | 3 | 1.60 | 1.76 | 0.2937 |
| Pure error | 3.64 | 4 | 0.91 | - | - |
| Correlation total | 165.26 | 16 | - | - | - |
| R^2^=0.9489 | **R^2^_Adj_**=0.8832 | **R^2^_Pred_**=0.5005 Adeq Precisior=11.340 | | | |

**Means significant differences (P<0.01).

As shown in **Figure S3-4** to **S3-6**, 3D response surfaces and 2D contour plots were generated, which shown the interaction of the variables and the optimal level of each variable for maximum response.


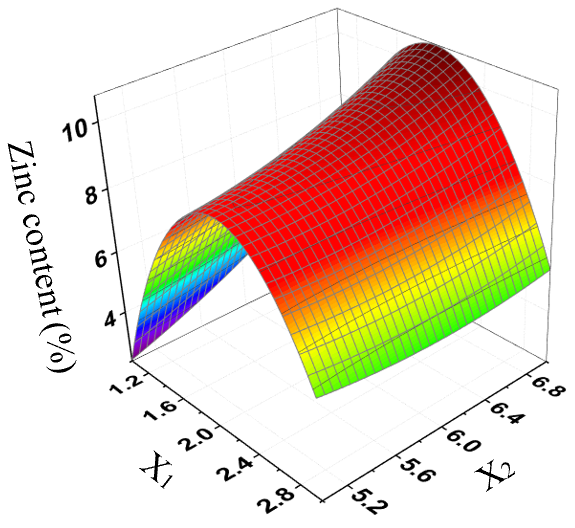


X_2_

X_1_


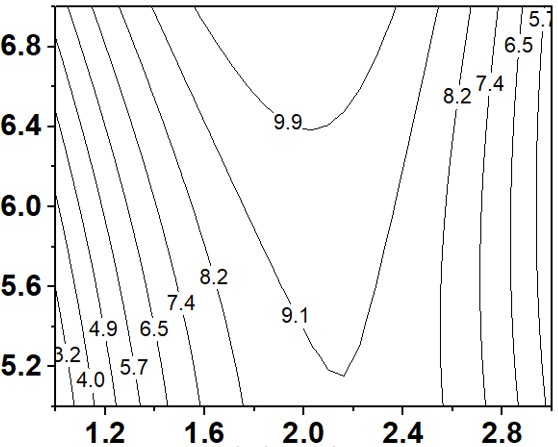


**Figure S3-4** The response surface plots of the effect of time(X_1_), pH(X_2_) and their reciprocal interaction on the zinc content of CMPZ (%).


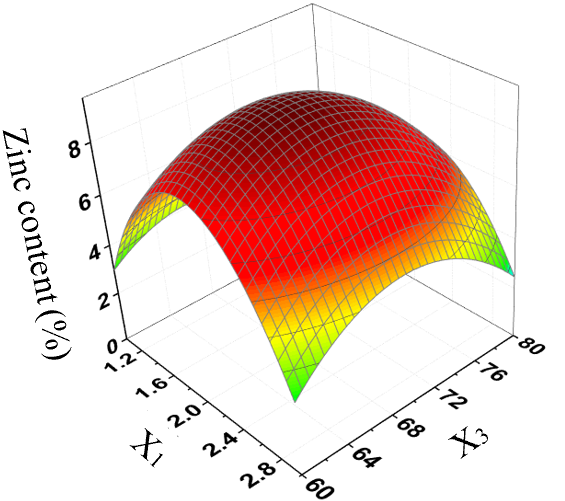


X_3_


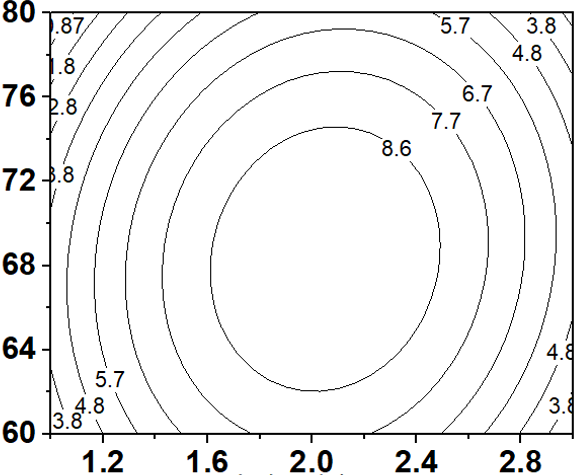


X_1_

**Figure S3-5** The response surface plots of the effect of time(X_1_), temperature(X_3_) and their reciprocal interaction on the zinc content of CMPZ (%).


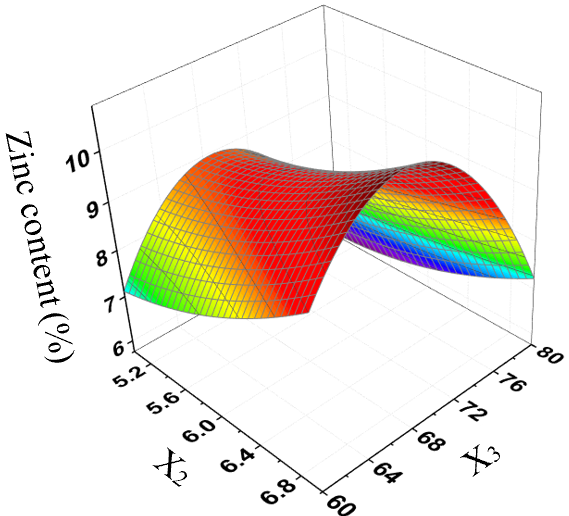


X_3_


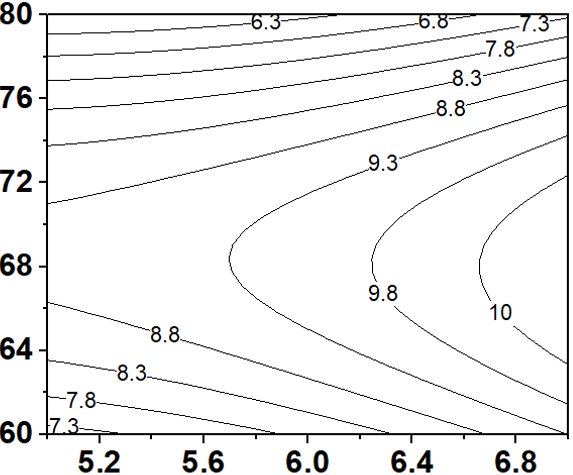


X_2_

**Figure S3-6** The response surface plots of the effect of pH(X_2_), temperature(X_3_) and their reciprocal interaction on the zinc content of CMPZ (%).

The interaction and influence of various factors in the response surface can be directly reflected by the contour density in the contour map and the steepness in the response surface map in **Figure** **S3-4 to S3-6**. It can be seen from **Figure** **S3-4 to S3-6** that there was a maximum value in the range of the highest point and contour line of the response surface, and the highest point in the figure was also the center point of the smallest ellipse in the contour line. As shown in the figure above, the contour map of CMPZ shows that the ellipse shows that the interaction between various factors was very strong, and the influence on the synthesis of CMPZ was significant.

**2.3 Validation of polysaccharide iron preparation optimization process**

According to these results, the optimal synthesis process conditions predicted by the response surface software were as follows: time 1.95h, a pH of 7 and the reaction temperature of 67.79°C. Under these optimal conditions, and the experimental zinc content of CMPZ was 10.41%. Compared with the predicted value of 10.85%, there was a 95.94% agreement, and the deviation rate was 4.06%, indicating that the response surface model is good and can be used to predict the iron content of CMPZ.
